# Asymmetrical distribution of non-conserved regulatory sequences at *PHOX2B *is reflected at the ENCODE loci and illuminates a possible genome-wide trend

**DOI:** 10.1186/1471-2164-10-8

**Published:** 2009-01-07

**Authors:** David M McGaughey, Zachary E Stine, Jimmy L Huynh, Ryan M Vinton, Andrew S McCallion

**Affiliations:** 1 McKusick – Nathans Institute of Genetic Medicine, Johns Hopkins University School of Medicine, 733 N. Broadway, BRB Suite 449, Baltimore, MD 21205, USA; 2 Department of Molecular and Comparative Pathobiology, Johns Hopkins University School of Medicine, Baltimore, MD 21205, USA

## Abstract

**Background:**

Transcriptional regulatory elements are central to development and interspecific phenotypic variation. Current regulatory element prediction tools rely heavily upon conservation for prediction of putative elements. Recent *in vitro *observations from the ENCODE project combined with *in vivo *analyses at the zebrafish *phox2b *locus suggests that a significant fraction of regulatory elements may fall below commonly applied metrics of conservation. We propose to explore these observations *in vivo *at the human *PHOX2B *locus, and also evaluate the potential evidence for genome-wide applicability of these observations through a novel analysis of extant data.

**Results:**

Transposon-based transgenic analysis utilizing a tiling path proximal to human *PHOX2B *in zebrafish recapitulates the observations at the zebrafish *phox2b *locus of both conserved and non-conserved regulatory elements. Analysis of human sequences conserved with previously identified zebrafish *phox2b *regulatory elements demonstrates that the orthologous sequences exhibit overlapping regulatory control. Additionally, analysis of non-conserved sequences scattered over 135 kb 5' to *PHOX2B*, provides evidence of non-conserved regulatory elements positively biased with close proximity to the gene. Furthermore, we provide a novel analysis of data from the ENCODE project, finding a non-uniform distribution of regulatory elements consistent with our *in vivo *observations at *PHOX2B*. These observations remain largely unchanged when one accounts for the sequence repeat content of the assayed intervals, when the intervals are sub-classified by biological role (developmental versus non-developmental), or by gene density (gene desert versus non-gene desert).

**Conclusion:**

While regulatory elements frequently display evidence of evolutionary conservation, a fraction appears to be undetected by current metrics of conservation. *In vivo *observations at the *PHOX2B *locus, supported by our analyses of *in vitro *data from the ENCODE project, suggest that the risk of excluding non-conserved sequences in a search for regulatory elements may decrease as distance from the gene increases. Our data combined with the ENCODE data suggests that this may represent a genome wide trend.

## Background

Transcriptional regulatory elements play a critical role in disease [1-3], development [4,5], and interspecific phenotypic variation [6-8]. However, unlike coding sequences, there is no universal vocabulary that can infer their biological relevance based on sequence composition alone, and, unlike structural RNAs, there is no associated secondary structure which can aid in their identification.

Comparative genomic sequence analysis has proven to be a powerful tool for the identification of putatively functional sequences, predicting functional significance based on evolutionary conservation [9-15]. A logical extrapolation of this approach has been to focus on highly or ultra-conserved non-coding elements as regions presumably enriched for regulatory sequences [9,16-18]. However, functional regulatory elements have also been identified using less stringent definitions of constraint [11,19-22]. Taken together these studies suggest that no single evolutionary distance can capture all functional regulatory elements. Consistent with this are reports of reduced promoter evolutionary constraint between human and chimpanzee [23], wide spread cis-regulatory element shuffling [24] and mammalian putative regulatory regions that do not align to other mammalian genomes [25-28]. Furthermore, we recently completed a comprehensive functional screen for regulatory activity by tiling sequences across the zebrafish *phox2b *locus and demonstrated that, at least at that locus, a significant fraction of regulatory sequences displayed no evidence of functional constraint [29]. However zebrafish and fugu, its most closely related sequenced genome, are substantially more evolutionarily separated than humans are from rodents (3–400 million years compared to 80–100 million years). Thus, one explanation for this observation is that functional non-conserved sequences are detectably conserved over shorter evolutionary distances. We directly address this idea by assaying sequences from a tiled interval of the human *PHOX2B *locus. We hypothesize that functional non-conserved sequences are likely present at the human locus in comparable frequency to the zebrafish *phox2b *ortholog [29]. *PHOX2B *is a three exon gene that spans approximately 4.9 kb and encodes a paired homeobox domain transcription factor. This developmentally critical gene is tightly regulated and mutations in its coding sequence have been implicated in several human pathologies, including central congenital hypoventilation syndrome, neuroblastoma, and Hirschsprung disease [30-34].

The recent public release of data from the ENCODE project [25] provided a large data set to explore putative regulatory element conservation and distribution. Using protein occupancy and chromatin modification data from the ENCODE project, 1,394 of the most likely transcriptional regulatory elements were identified and termed putative transcriptional regulatory regions (pTRRs) [26]. Consistent with our observations at *phox2b*, the ENCODE consortium reports a markedly low coincidence between pTRRs and multi-species constrained sequences [25,26,35,36]. While the enrichment of putative regulatory elements proximal to coding regions [35] has similarly been noted, the relative distribution of non-conserved proximal and distal intergenic pTRRs has not to our knowledge been examined thoroughly.

We set out to address three main questions using a transposon-based transgenic approach in zebrafish embryos. First, we tested whether the human *PHOX2B *locus contained functional non-conserved regulatory sequences in addition to conserved *PHOX2B *functional elements, similar to its zebrafish ortholog. We assayed the regulatory potential of a tiled interval encompassing ten kilobases (kb) upstream of *PHOX2B *as well as the intronic sequences. Second, we assayed human *PHOX2B *non-coding sequences across a 135 kb interval that were conserved with previously identified zebrafish *phox2b *regulatory sequences, demonstrating that most overlap in their regulatory control; the functionality of these human to zebrafish orthologous sequences remains generally unchanged with increasing distance from the gene. Third, by using these regulatory sequences as ''anchors,'' we tested flanking sequences that were not conserved and provide preliminary evidence of a trend that non-conserved regulatory sequences tend to lie more frequently proximal to the *PHOX2B/phox2b *exons. To test if this apparent non-uniform distribution of non-conserved regulatory elements represents a genome-wide characteristic, we determined the distribution of the non-conserved pTRRs reported for the ENCODE intervals [26] and demonstrated the concordance between our data and the ENCODE-derived data.

## Results

### Conserved and non-conserved *PHOX2B *non-coding elements direct expression

**Figure 1:**
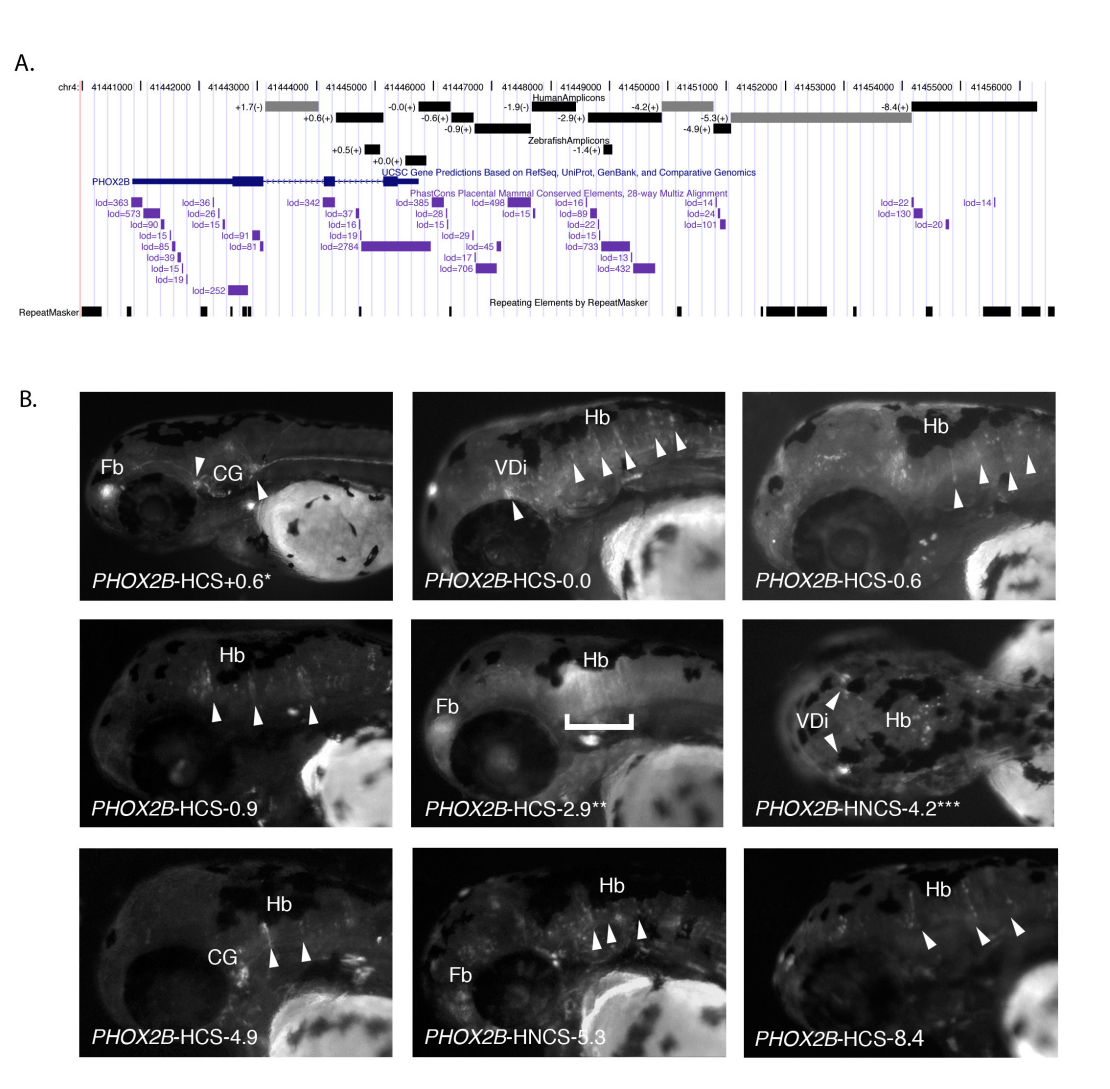
**Conserved and non-conserved amplicons tiled across the *PHOX2B *proximal region direct *PHOX2B *appropriate expression**. (a) The human *PHOX2B *promoter proximal region (chr4:41,440,000–41,456,600; hg18) was divided into 11 amplicons (total size 11,965 base pairs) excluding exons, 5' UTR, and 3' UTR, according to whether intervals contained PhastCons Placental Mammal Conserved Elements, 28-way Multiz Alignment sequences [41]. The amplicons are represented as gray scale rectangles: black (*PHOX2B*-HCS); gray (*PHOX2B*-HNCS); black (zebrafish alignment). Amplicon names are defined by their distance from the *PHOX2B *transcriptional start site and are displayed as custom tracks on the UCSC Genome browser [54] (b) Lateral images of G0 48-hpf zebrafish embryos exhibiting *PHOX2B *appropriate expression with element name marked on picture. Fb, Forebrain; VDi, Ventral Diencephalon; Hb, Hindbrain; CG, Cranial Ganglia; SC, Spinal Cord; ENS, Enteric Nervous System. *G1 embryo at 72-hpf. **G1 embryo at 48-hpf. *** Dorsal photo.

**Figure 2:**
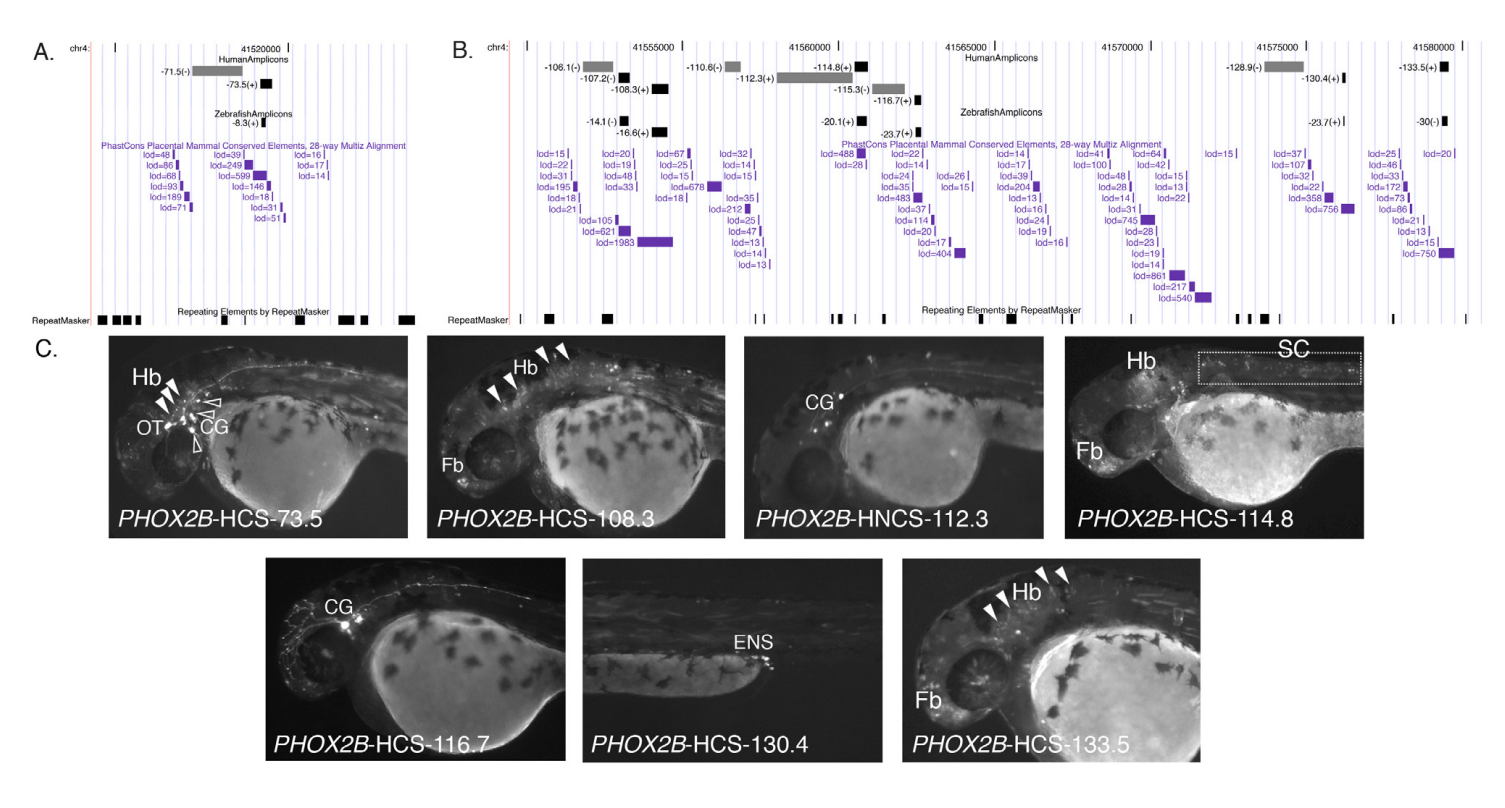
***PHOX2B *human distal conserved sequences demonstrate activity consistent with orthologous zebrafish sequences**. The interval displayed as a custom track on UCSC Genome browser [54]. The amplicons are represented as gray scale rectangles: black (conserved), gray (non-conserved), black (zebrafish alignment). (A) Region containing region aligning to *phox2b*-ZCS -8.3 (chr4:41,516,361–41,521,080; hg18) (B) Region containing aligning to *phox2b*-ZCS -16.6,*phox2b-ZCS-20.1, phox2b*-ZCS -23.7 and *phox2b*-ZCS-30.0. (chr4:41,549,434–41,580,142; hg18) (C) Lateral images of G0 transgenic zebrafish embryos corresponding to functional human conserved (*PHOX2B*-HCS -73.5, *PHOX2B*-HCS -108.3, *PHOX2B*-HCS-114.8, *PHOX2B*-HCS -116.7, *PHOX2B*-HCS -130.4 and *PHOX2B*-HCS-133.5), and human non-conserved (*PHOX2B*-HNCS -112.3) amplicons. Fb, Forebrain; OT, Oculomotor and Trochlear Motor Progenitors; Hb, Hindbrain; CG, Cranial Ganglia; SC, Spinal Cord; ENS, Enteric Nervous System. Closed arrow-heads point to hindbrain expression. Open arrow-heads point to cranial ganglia expression.

We recently demonstrated that both conserved and non-conserved sequences tiled across the zebrafish *phox2b *locus frequently displayed regulatory control consistent with endogenous *phox2b *expression [29]. We, and others, have pointed out that the increased divergence among teleosts, compared with divergence among mammals, increases the likelihood that zebrafish functional sequence modules will have acquired additional substitutions, reducing overt conservation. Additionally, despite the duplicate presence of approximately 30% of genes in zebrafish, its genome is significantly more compact than mammalian genomes ([37,38]; 1.9 gigabase pairs in zebrafish versus 3.1 gigabase pairs in human genome). Consequently functional sequences therein likely exist in proportionally reduced sequence space. Taken together, these observations suggest that one is more likely to find functional non-conserved regulatory elements in teleosts than in mammals. However, it remains untested, until now, whether these observations can be wholly accounted for by the above explanations or whether they, in part, are representative of a broader trend in vertebrate genomes. We set out to determine whether the human *PHOX2B *locus contains non-conserved functional sequences like its zebrafish ortholog. In our recent study of *phox2b*, the non-coding sequence intervals proximal to the *phox2b *exons revealed more non-conserved regulatory sequences than those at distance of 10 kb or greater. Thus we postulated that, if they exist, human non-conserved *PHOX2B *regulatory elements would be identified most readily in the gene proximal region. We initially generated 11 Tol2-based transgenic constructs tiling across all non-coding sequences contained within the *PHOX2B *introns (two intronic constructs) as well as those contained within an interval of 10 kb 5' to the transcriptional start site (TSS) (nine 5' constructs). Synteny between mammalian and teleost *PHOX2b*/*phox2b *orthologs begins to break down 3' to the coding exons; thus we focused on intronic sequences and sequences 5' to the gene. We introduced each construct into ≥200 G0 zebrafish embryos as described previously [19,39] and assayed their ability to drive enhanced GFP (eGFP) reporter expression in cell populations consistent with *phox2b *during development. Although we, and others, have previously demonstrated that G0 analyses can provide a robust assay of regulatory elements when requiring concordant expression among many independent embryos [15,19,39,40], we also recognize that such analyses can over look some signals that become obvious only upon transmission through the germ line. In these analyses we sought to limit the potential for such events by requiring consistent signal among ≥10% of injected embryos; these embryos were used to score for function. Only elements that drove reproducible tissue specific expression were considered regulatory elements; images of representative embryos are provided for each identified regulatory element (Figure 1; Figure 2). Although the selected G0 images demonstrate that identified regulatory elements directed expression in cell types overlapping the endogenous *phox2b *expression domains we cannot definitively attribute the extent of their regulatory control nor exclude the potential for regulatory control beyond those domains. We can, however, identify that they display concordant functions as regulatory elements.

In our recent analysis of the zebrafish *phox2b *locus, we demonstrated that among the many commonly used sequence conservation algorithms, phastCons performed with the highest sensitivity and specificity [29,41]. Thus in our comparisons of the functionality of conserved versus non-conserved sequences, we classified amplicons based on overlap with phastCons Placental Mammal Conserved Elements, 28-way Multiz Alignment intervals [41-43]. The amplicons generated in this experiment comprised a tiling path of nine non-intronic elements (seven conserved, two non-conserved), encompassing 10,244 bp of the total 10,562 bp in this region, and 1,721 bp of sequence contained within its two introns (one conserved, one non-conserved; Figure 1A; Additional file 1 Table S1).

As expected, seven of the eight human conserved sequences (HCS), within 10 kb of *PHOX2B*, directed tissue-specific expression consistent with that of endogenous *phox2b*, including expression in neuronal populations of the ventral diencephalon (VDi), cranial ganglia (CG), and hindbrain (Hb)(Figure 1B). Additionally, two out of three assayed human non-conserved sequences (HNCS) in this interval also drove *phox2b*-appropriate expression, consistent with observations in zebrafish. *PHOX2B*-HNCS-4.2 directed expression in the ventral diencephalon and the hindbrain, while *PHOX2B*-HNCS-5.3 directed expression in the forebrain and hindbrain (Figure 1B). By contrast *PHOX2B*-HNCS+1.7 failed to direct detectable reporter expression during the developmental time points analyzed (Table 1). Importantly, these data are consistent with our previous findings at the orthologous *phox2b *zebrafish locus [29] and are consistent with our underlying hypothesis.

**Table 1:** Functional non-conserved elements exhibit non-uniform distribution.

Functional non-conserved sequences	Zebrafish *phox2b*	Human *PHOX2B*
≤ 10 kb from gene	3/5 functional	2/3 functional
>10 kb from gene	1/8 functional	1/6 functional

The distribution and function of tested non-conserved elements at the zebrafish *phox2b *locus [29] and human *PHOX2B *locus are detailed; elements are grouped according to position (less than or greater than 10 kb of the *PHOX2B *gene region).

### Orthologous *PHOX2B/phox2b *amplicons display overlapping functions

Since *PHOX2B *and its zebrafish ortholog are critical for neuronal development in both mammals and teleosts [33,44], we posited that the corresponding human and zebrafish orthologous non-coding sequences in this interval would share largely overlapping functions. The assayed human tiling path amplicons included three elements (*PHOX2B*-HCS+0.6, *PHOX2B*-HCS-0.0, *PHOX2B*-HCS-2.9) conserved to zebrafish; their orthologous conserved sequences (ZCS) are *phox2b*-ZCS+0.5, *phox2b*-ZCS+0.0 and *phox2b*-ZCS-1.4 (Table 2). *PHOX2B*-HCS-0.0 directed expression in the ventral diencephalon and hindbrain in G0 fish (Figure 1B), overlapping with the hindbrain expression observed in the previously published G1 results using the orthologous zebrafish sequence (Table 2 and Additional file 1 Table S1; [29]). *PHOX2B*-HCS-2.9 directed reporter expression in the forebrain and hindbrain, overlapping the hindbrain reporter expression directed by its zebrafish ortholog (*phox2b*-ZCS-1.4; [29]). Furthermore, although *PHOX2B*-HCS+0.6 drove reporter expression in the forebrain and cranial ganglia consistent with *phox2b*, these sites also extend beyond the observed regulatory control of the orthologous zebrafish *phox2b*-ZCS+0.5 sequence. The incomplete nature of overlap in regulatory control observed for these elements may reflect lineage-specific adaptation of a common ancestral functional element (Figure 1B, Table 1, Table 2). Importantly, analyses of *PHOX2B*-HCS+0.6 and *PHOX2B*-HCS+2.9 in G0 were validated by passage through the germ line and analysis of = 2 independent lines per construct. The resulting observations (Figure 1B) were consistent between G0 and G1 embryos analyzed.

**Table 2:** Human *PHOX2B *elements conserved to zebrafish *phox2b *locus demonstrate activity consistent with orthologous zebrafish sequences.

Human Amplicon	Expression (HCS)	Zebrafish Amplicon	Expression (ZCS)	Coincident Control
*PHOX2B*-HCS+0.6*	Fb, CG	*phox2b*-ZCS +0.5	Hb, SC	No
*PHOX2B*-HCS -0.0	VDi, Hb	*phox2b*-ZCS +0.0	Hb, SC	Yes
*PHOX2B*-HCS -2.9*	Fb, Hb	*phox2b*-ZCS -1.4	Mb, Hb, SC	Yes
*PHOX2B*-HCS -73.5	OT, CG, Hb	*phox2b*-ZCS -8.3	Mb, Hb, CG	Yes
*PHOX2B*-HCS -108.3	Fb, Hb	*phox2b*-ZCS -16.6	Mb, Hb	Yes
*PHOX2B*-HCS-114.8	Fb, Hb, SC	*phox2b*-ZCS-20.1	Hb	Yes
*PHOX2B*-HCS -116.7	CG	*phox2b*-ZCS -23.7	Hb, SC, ENS	No
*PHOX2B*-HCS -130.4	ENS	*phox2b*-ZCS -23.7	Hb, SC, ENS	Yes

HCS expression is pattern driven by human amplicon in G0 zebrafish embryos (* indicates G1 expression pattern). ZCS expression is pattern driven by orthologous zebrafish amplicon in G1 zebrafish embryos [29]. Overlap is categorized as Yes, tissue overlap in expression patterns, but additional tissues seen; No, no overlap in expression. Fb, Forebrain; OT, Oculomotor and trochlear motor Progenitors; VDi, Ventral diencephalon; Hb, Hindbrain; CG, Cranial ganglia,; SC, Spinal cord ENS, Enteric nervous system.

To further test our hypothesis, we expanded our analyses of human *PHOX2B *sequences orthologous to the remaining four previously identified *phox2b *zebrafish enhancers (*phox2b*-ZCS -8.3, *phox2b*-ZCS-16.6, *phox2b*-ZCS-20.1 and *phox2b*-ZCS-23.7) that align to the human *PHOX2B *locus [29]. These sequences were scattered over an interval in excess of 130 kb 5' to the *PHOX2B *gene. Zebrafish sequence elements *phox2b*-ZCS-8.3, *phox2b*-ZCS-16.6 and *phox2b*-ZCS-20.1 aligned with the human sequences *PHOX2B*-HCS-73.5, *PHOX2B*-HCS-108.3 and *PHOX2B*-HCS-114.8, respectively (Figure 2A, 2B; Table 2; Additional file 1 Table S1). However, the zebrafish amplicon *phox2b*-ZCS-23.7, which represents a tight cluster of highly conserved sequence intervals (4.2 kb) aligned to human genomic sequences scattered over 13.8 kb, more than 115 kb 5' to the human *PHOX2B *TSS. We selected intervals *PHOX2B*-HCS-116.7 and *PHOX2B*-HCS-130.4, aligning to *phox2b*-ZCS-23.7, as representative sequences for evaluation (Figure 2B, Table 2 and Additional file 1 Table S1).

*PHOX2B*-HCS-73.5 directed reporter expression in the oculomotor and trochlear motor progenitors, cranial ganglia and hindbrain, consistent with the cranial ganglia and hindbrain reporter expression pattern exhibited by the zebrafish orthologous element *phox2b*-ZCS-8.3 (Figure 2A, 2C) [29]. However, in contrast with its zebrafish ortholog, *PHOX2B*-HCS-73.5 did not direct detectable expression in the midbrain. As noted above, this may reflect lineage-specific adaptation of the common ancestral regulatory sequences by the zebrafish and human *phox2b/PHOX2B *loci. It may also reflect, in part, the mosaic nature of the reporter expression observed in G0 embryos. However, one might reasonably expect that cell populations present in lower abundance within the embryo might be more likely to be overlooked in mosaics and not *vice versa*. Amplicon *PHOX2B*-HCS-108.3 directed reporter expression in the forebrain and hindbrain, overlapping the hindbrain expression its orthologous zebrafish sequence directed (*phox2b*-ZCS-16.6; Figure 2C, Table 2 and Additional file 1 Table S1, [29]). Moving more distal from the *PHOX2B *coding sequence, *PHOX2B*-HCS-114.8 directed expression in the forebrain, hindbrain, and spinal cord, once again overlapping the tissue specific hindbrain regulatory activity of its zebrafish orthologous sequence (*phox2b*-ZCS-20.1; Figure 2C; Table 2; [29]). Interestingly, although *PHOX2B*-HCS-116.7 was functionally active within the central nervous system, its tissue-specific regulatory control was discrete from that of *phox2b*-ZCS-23.7, to which it aligns. *PHOX2B*-HCS-116.7 directed expression within cranial ganglia, contrasting with the hindbrain, spinal cord and enteric nervous system expression demonstrated by the orthologous zebrafish sequence *phox2b*-ZCS-23.7 (Figure 2C, Table 2; [29]). Despite lying immediately proximal to an uncharacterized primate-specific predicted gene,*PHOX2B*-HCS-130.4 directed enteric nervous system-specific reporter expression overlapping the expression pattern displayed by *phox2b*-ZCS-23.7 (Figure 2C, Table 2 and Additional file 1 Table S1, [29]). These data are largely consistent with our underlying hypothesis that orthologous sequences drive overlapping expression patterns. Overall, six of the eight enhancer sequences that aligned between zebrafish and human overlapped in their regulatory control; these observations are, of course, consistent with their conservation and with their potentially important role in vertebrate nervous system development.

We then tested three human regions at the *PHOX2B *locus (*PHOX2B*-HCS-34.8, *PHOX2B*-HCS-107.2 and *PHOX2B*-HCS-133.5) that aligned to conserved zebrafish *phox2b *sequences lacking detectable enhancer function (*phox2b*-ZCS-3.5, *phox2b*-ZCS-14.1, and *phox2b*-ZCS-30.0, respectively) in our previous analysis (Figure 2A, 2B, [29]). Consistent with their zebrafish orthologs, neither *PHOX2B*-HCS-34.8 nor *PHOX2B*-HCS-107.2 exhibited detectable regulatory control. *PHOX2B*-HCS-133.5, however, did display regulatory control, directing reporter expression in the forebrain and hindbrain (Figure 2C). Once again this observation may represent lineage-specific adaptation of an ancestral regulatory element; we also cannot exclude the possibility that the zebrafish element (*phox2b*-ZCS-30.0) may function at times outside the window examined (24–96 hour post fertilization). Consistent with our initial postulate, 8 of 11 assayed human sequences displayed regulatory control overlapping their zebrafish orthologs. Perhaps unsurprisingly, 8 of 11 human sequences conserved to zebrafish also displayed some spatial control within the nervous system that was absent from their zebrafish orthologs, consistent with lineage-specific variation in regulatory control.

### *in vivo *functional validation of skewed non-conserved regulatory element distribution at *phox2b/PHOX2B*

We posited that non-conserved regulatory elements occur less frequently with an increasing distance from the gene. Further evaluation of our recent analysis at the zebrafish *phox2b *locus [29] suggests that three of five zebrafish non-conserved (ZNCS) regulatory elements within 10 kb of the gene exhibit function. Zebrafish elements *phox2b*-ZNCS+6.7, *phox2b*-ZNCS+5.6 and *phox2b*-ZNCS+3.1 drove *phox2b *consistent expression, while *phox2b*-ZNCS-4.9 and *phox2b*-ZNCS-5.9 did not exhibit regulatory activity [29]. However, only one of eight non-conserved zebrafish amplicons greater than 10 kb from the gene exhibited regulatory activity (*phox2b*-ZNCS-27.9; Table 1; [29]). We posited that the human *PHOX2B *locus might exhibit the same characteristic distribution of non-conserved regulatory elements (Table 1).

To directly address this idea, we assayed six additional non-conserved sequences flanking functional distal regulatory elements conserved from zebrafish to human (Figure 2A–C, Additional file 1 Table S1). The non-conserved element *PHOX2B*-HNCS-71.5, which is adjacent to *PHOX2B*-HCS-73.5 (Figure 2a), exhibited no function upon injection of ≥200 G0 zebrafish embryos. Upon injection and assay of comparable numbers of embryos with constructs *PHOX2B*-HNCS-106.1, *PHOX2B*-HNCS-110.6, and *PHOX2B*-HNCS-112.3 (Figure 2B), only *PHOX2B*-HNCS-112.3 drove expression. This element directed reporter expression in the cranial ganglia, consistent with endogenous *phox2b *expression (Figure 2C). Additionally, we selected and analyzed elements *PHOX2B*-HNCS-115.3 and *PHOX2B*-HNCS-128.9, immediately flanking the conserved enhancers *PHOX2B*-HCS-116.7, *PHOX2B*-HCS-130.4, respectively; both non-conserved amplicons failed to direct reproducible reporter expression in any tissues at all time points examined (Figure 2B). Thus, of these six human distal non-conserved sequences tested, only *PHOX2B*-HNCS-112.3 displayed evidence of tissue-specific regulatory control (Table 1; Figure 2A–C). These data are consistent with our earlier observations at the zebrafish *phox2b *locus (Table 1; [29]). If one examines the corresponding human and zebrafish data sets for sequences within 10 kb of the gene, they similarly suggest a bias in the distribution of functional non-conserved, non-coding sequences proximal to the gene. Comparison of the data generated at the human and zebrafish *PHOX2B/phox2b *orthologs demonstrate that two of the three non-conserved elements tested in the *PHOX2B *(TSS-proximal) tiling path drove expression consistent with endogenous *phox2b *expression (Figure 1; [29]), which agrees with the data generated at *phox2b *in zebrafish, for the same interval (Table 1; [29]).

While it remains possible that the observed skewed distribution of non-conserved, functional non-coding sequences is unique to *PHOX2B/phox2b *or represents an artifact of a small sample size, we posited that it might also represent a more general characteristic of vertebrate genomes. When combining the zebrafish and human data, five of eight non-conserved elements within 10 kb of the gene exhibit regulatory function, while only two of 14 non-conserved gene distal elements exhibit regulatory function (Table 1; Figure 1; Figure 2; [29]). Taken together these data suggest that the non-conserved elements proximal to genes may have an increased probability of functioning as a transcriptional regulatory element. These *in vivo *observations at the *PHOX2B *locus indicate a potential trend that functional non-conserved regulatory sequences may not be uniformly distributed with respect to genes; functional non-conserved amplicons display a slightly skewed distribution, with a higher frequency more proximal to genes and a lower frequency in more distal regions. We sought to test the generality of these observations to other loci using the recently published ENCODE data [25].

### The non-uniform distribution of non-conserved regulatory sequences represents a genome-wide phenomenon

Until the completion of the human, mouse and other vertebrate genome sequences, the 5' putative promoter regions of genes were the primary site of inquiry for vertebrate regulatory sequences. Although existing functional data sets of regulatory sequences may consequently be enriched for these sequence intervals, the recently completed ENCODE project also reports a similar trend [25,26,35]. Prompted by the observation of a potential unequal distribution of conserved regulatory elements at the *PHOX2B *locus, we set out to determine whether the distribution of non-conserved regulatory elements also contributes to this genome wide trend. King and colleagues [26] recently identified 1,394 putative transcriptional regulatory regions (pTRRs) within the 1% of the human genome evaluated by ENCODE [25]. We examined this data, classifying pTRRs as conserved or non-conserved based upon whether they overlapped with PhastCons Placental Mammal Conserved Elements, 28-way Multiz Alignment intervals [10,41,43]. We then applied the ENCODE defined sub-region identifiers (coding sequence, 5' UTR, 3' UTR, intronic proximal, intronic distal, intergenic proximal, or intergenic distal) to the pTRRs (Additional file 1 Table S2, Table 3). As with our analysis of non-conserved sequences at the *PHOX2B *and *phox2b *loci, we focused on pTRRs within intergenic proximal (intervals closer than 5 kb to nearest exon) and intergenic distal intervals (farther than 5 kb from the nearest exon). After calculating the total number of conserved and non-conserved base pairs in each sub-region (see Methods for details on calculations), we then determined the density of non-conserved pTRRs within intergenic proximal and intergenic distal sub-regions (Table 4, Additional file 1, Table S2). Non-conserved pTRR density was defined as the number of non-conserved pTRRs in the region per non-conserved base pair in the region. Interestingly, the density of non-conserved pTRRs within intergenic proximal sub-regions was 4.33 fold higher than the density within intergenic distal sub-regions (Table 4, Table 5). Although potentially interesting, we also noted that these observations could be significantly confounded by several factors; we thus addressed each in turn.

**Table 3:** Distribution of putative transcriptional regulatory regions (pTRRs) identified by King et al. [26].

Type of gene	ENCODE Sub-regions Analyzed	Conserved pTRRs in sub-region	Non-conserved pTRRs in sub-region	Base pairs in sub-region
All	5' UTR	71	46	99,440
All	3' UTR	15	12	382,329
All	Intergenic Proximal	61	163	2,429,196
All	Intergenic Distal	48	171	11,055,834
All	Intronic Proximal	173	457	8,903,959
All	Intronic Distal	55	122	6,462,925
All	Coding sequences	0	0	671,166
Developmental	Intergenic Proximal	20	22	392,692
Developmental	Intergenic Distal	5	20	1,636,075
Non-developmental	Intergenic Proximal	24	86	733,487
Non-developmental	Intergenic Distal	10	51	2,309,353
Non-gene Desert	Intergenic Distal	39	159	7,147,316
Gene Desert	Intergenic Distal	9	12	3,908,518
Gene Desert	Intergenic Proximal	0	3	75,000
Non-gene Desert	Intergenic proximal	61	160	2,354,196

pTRRs partitioned to ENCODE defined regions and grouped as conserved versus non-conserved based on pTRR overlap with PhastCons Placental Mammal Conserved Elements, 28-way Multiz Alignment [43]. Gene type of all represents analysis of whole ENCODE region. Developmental genes represent regions flanking genes labeled with Gene Ontology term GO:0032502, while non-developmental genes were those that were not labeled with GO:0032502. Gene deserts were ENCODE intervals overlapping regions ≥500 kb without a Reference Sequence gene. Non-gene deserts were all sub-regions that did not overlap gene deserts. The "Base pairs in sub-region" column represents the sum of the genomic intervals represented by each type of sub-regions.

**Table 4:** Non-conserved pTRR density is higher in intergenic proximal regions than intergenic distal regions.

		pTRR Density			
		
Gene Type	ENCODE Sub-region	Conserved pTRRs/Conserved bp	Non-conserved pTRRs/Non-conserved bp	Conserved pTRRS/Conserved Non-repeat bp	Non-conserved pTRRs/Non-conserved Non-repeat bp
All	5' UTR	1/457	1/1,456	1/410	1/931
All	3' UTR	1/5,625	1/24,829	1/5,460	1/18,610
All	Intergenic Proximal	1/1,185	1/14,460	1/1,094	1/7,005
All	Intergenic Distal	1/7,528	1/62,541	1/7,073	1/29,635
All	Intronic Proximal	1/1,463	1/18,930	1/1,356	1/10,801
All	Intronic Distal	1/4,259	1/51,055	1/4,026	1/28,231
Developmental	Intergenic Proximal	1/1,591	1/16,403	1/1,508	1/8,796
Developmental	Intergenic Distal	1/11,370	1/78,961	1/10,714	1/47,792
Non-developmental	Intergenic Proximal	1/867	1/8,287	1/821	1/3,619
Non-developmental	Intergenic Distal	1/6,160	1/44,074	1/5,776	1/18,446
Non-gene Desert	Intergenic Proximal	1/1,078	1/14,303	1/991	1/6,877
Gene Desert	Intergenic Proximal	N/A	1/22,824	N/A	1/13,830
Non-gene Desert	Intergenic Distal	1/5,307	1/43,650	1/4,954	1/19,047
Gene Desert	Intergenic Distal	1/17,151	1/312,847	1/16,254	1/151,566

Density of putative transcriptional regulatory regions (pTRRS) identified by King et al. [26]. The ENCODE interval was partitioned into sub-regions. Gene type of all represents analysis of whole ENCODE region. Developmental genes represent regions flanking genes labeled with Gene Ontology term GO:0032502, while non-developmental genes were those that were not labeled with GO:0032502. Gene deserts were ENCODE intervals overlapping ≥500 kb regions without a Reference Sequence gene. Non-gene deserts were all sub-regions that did not overlap gene deserts. Density of pTRRs was calculated by dividing the total number of conserved or non-conserved base pairs in the ENCODE defined region by number of conserved or non-conserved pTRRs in the ENCODE defined region. N/A= not applicable due to lack of conserved pTRRs in gene desert intergenic proximal regions

**Table 5:** Fold change in pTRR density of intergenic versus intergenic distal regions.

	Fold change of pTRR density between Intergenic Proximal and Intergenic Distal Sub-regions			
	
Gene Type	Conserved pTRRs/ Conserved bp	Non-conserved pTRRs/ Non-conserved bp	Conserved pTRRs/Conserved Non-repeat bp	Non-conserved pTRRs/ Non-conserved Non-repeat bp
All	6.35	4.33	6.47	4.23
Developmental	7.15	4.81	7.10	5.43
Non-developmental	7.10	5.32	7.04	5.10
Gene Desert	N/A	13.71	N/A	10.96
Non-gene Desert	4.92	3.05	5.00	2.77

Gene type of all represents analysis of whole ENCODE region. Developmental genes represent regions flanking genes labeled with Gene Ontology term GO:0032502, while non-developmental genes were those that were not labeled with GO:0032502. Gene deserts were ENCODE intervals overlapping ≥500 kb regions without a reference sequence gene. Non-gene deserts were all sub-regions that did not overlap gene deserts. Fold change in gene density was calculated for each gene type by dividing the intergenic proximal pTRR density by the intergenic distal pTRR density (Table 4). The pTRR densities were calculated for conserved pTRRs divided by conserved bp, non-conserved pTRRs divided by non-conserved bp, conserved pTRRs divided by conserved non-repeat bp and non-conserved pTRRs divided by non-conserved non-repeat bp (Table 3). N/A = not applicable due to lack of conserved pTRRs in gene desert intergenic proximal regions.

First, much of the genome is composed of repeats whose distribution is non-uniform, we thus set out to determine if the difference in non-conserved pTRR density between intergenic proximal and intergenic distal regions was due to density of repetitive DNA elements within evaluated intervals. We then calculated the total number of conserved and non-conserved repeat base pairs in each sub-regions, which where then used to calculate the number of conserved non-repeat base pairs and non-conserved non-repeat base pairs in each sub-region (Additional file 1, Table S2, See methods for details of calculations). Adjusted for repeats, the density of non-conserved pTRRs is 4.23 times higher in intergenic proximal regions than in intergenic distal sub-regions, suggesting that the trend observed of higher non-conserved pTRR density proximal to the gene compared to distal to the gene is not due to a difference in repetitive element density in the sub-regions (Table 4; Table 5; Additional file 1 Table S2).

Second, developmental genes are reported to require more regulatory control modules than non-developmental genes [45-47]. We therefore asked whether the density of pTRRs differed between sequences flanking developmental and non-developmental genes. We utilized Gene Ontology (GO) to differentiate between developmental and non-developmental genes, using GO term GO:0032502 to define developmental genes [48]. Using "GO Slimmer" ; [49]), 78 unique genes from the ENCODE intervals labeled with GO:0032502 were identified as developmental genes, while 152 genes from the ENCODE region were correspondingly identified as non-developmental. The fold change in densities of non-conserved pTRRs from intergenic proximal to intergenic distal for developmental genes (4.81 fold change in density) and non-developmental (5.32 fold change in density) were consistent with those in analysis of all ENCODE intergenic proximal and intergenic distal sub-regions (4.32 fold change in density; Table 4; Table 5). Additionally, the fold change in density between the intergenic proximal and intergenic distal sub-regions when adjusting for repeats remained similar for both the developmental genes (5.43 fold change in density) and non-developmental genes (5.10 fold change in density; Additional file 1, Table S3; Table 4; Table 5).

Finally, we sought to determine whether our observations still held true in comparisons of gene desert intervals alone and those specifically excluding gene deserts. To examine the effect of gene deserts on pTRR density, we compared gene desert intergenic proximal and intergenic distal sub-regions to non-gene desert intergenic proximal and intergenic distal regions. Intergenic distal sub-regions were manually curated to identify the sub-regions that overlapped gene deserts, defined as intervals ≥500 kb without a National Center for Biotechnology Information Reference Sequence gene [50,51]. After analysis was performed as described above on the gene desert and non-gene desert sub-regions (Additional file 1, Table S4), the pTRR densities were once again calculated (Table 4). The gene desert intergenic distal non-conserved pTRR density was nearly an order of magnitude (7.17 fold) lower (1 non-conserved pTRR per 312,847 non-conserved base pairs) than that of non-gene desert intergenic distal regions (1 non-conserved pTRR per 43,650 base pairs), consistent with our underlying hypothesis that as distance from the gene increases, the density of non-conserved regulatory elements decreases. However, since gene desert flanking intergenic proximal regions represent such a small fraction (0.25%) of the total base pairs analyzed (Additional file 1 table S4), it is difficult to draw any conclusions on pTRR density for this subset alone. However, we can say that when comparing non-gene desert intergenic proximal regions to non-gene desert intergenic distal regions, we still observe a 3.02 fold change in non-conserved pTRR density. These data indicate that while gene deserts may contribute to the skewed pTRR density, there remains a significant fraction that cannot be accounted for by their effect.

## Discussion

The identification of functional non-coding regulatory sequence relies heavily upon commonly applied metrics of constraint. However, our previous analysis at the zebrafish *phox2b *locus identified numerous functional non-coding regulatory sequences that were not under any detectable constraint [29], raising into question the frequency with which current approaches overlook functional elements. However, the large evolutionary distance between teleosts reduces the ability to detect constraint, so we undertook a similar analysis using the human *PHOX2B *locus.

The human *PHOX2B *and zebrafish *phox2b *gene proximal regions exhibited similar densities of both conserved and non-conserved functional elements, suggesting that our original observations at the zebrafish *phox2b *locus may not simply be an artifact of the increased evolutionary distance among teleosts compared with mammals. Furthermore, the numbers of non-conserved functional regulatory sequences decreased with increasing distance from the gene for both the zebrafish and human *phox2b/PHOX2B *loci. We and others have proposed that the existence of non-conserved regulatory elements may result from lineage specific regulatory elements, transcription factor binding site shuffling, or elements falling below the threshold of detectable conservation [23,24,26,29]. Consistent with these postulates, analyses among more closely related species may increase the sensitivity and decrease the specificity with which one identifies true functional elements based upon conservation alone.

While previous studies have commented on the enrichment of regulatory elements proximal to genes [25,26,35], the relative distribution of non-conserved regulatory elements had not been assessed in detail. We directly addressed this question *in vivo *and, using the ENCODE data set, *in silico*. Our analysis of ENCODE-defined pTRRs [25,26] is consistent with the *in vivo *data both presented here for human *PHOX2B *and published previously for the zebrafish *phox2b *ortholog [29], as well as with previously published predictions from cell line derived data [27]. We demonstrated that ENCODE-identified pTRRs [26] lacking conserved sequence intervals defined by phastCONS were present at a 4.33 fold higher density in intergenic proximal regions compared to the intergenic distal region (Table 4, Table 5). These trends were maintained even when accounting for repeat density or class-specific biases associated with developmental versus non-developmental genes. Also consistent with our underlying hypothesis, we noted a lower density of intergenic distal pTRRs in gene desert regions than in non-gene desert regions. This observation is also consistent with data generated by Roh et al. [27], who reported a similar enrichment of proximal non-conserved putative enhancers identified by histone acetylation patterns in vitro. Collectively these data suggest that the fraction of regulatory elements that can be detected by conservation alone may be smaller than previously believed.

## Conclusion

The human and zebrafish *PHOX2B/phox2b *data taken in combination with previously published data [27] and our analysis of the publicly available ENCODE data [25,26] suggests that although conservation is a robust strategy to find functional sequences, implementation of this strategy alone will potentially overlook significant numbers of functional elements, particularly in regions proximal to genes. Importantly, these findings may significantly impact the search for regulatory variation underlying disease risk. The data suggest that although sequence conservation is a valid and often informative starting point for the identification of biologically functional sequences, there are frequently functional sequences that lie beneath that predictive radar. These observations suggest that the risk of overlooking non-conserved regulatory sequences at this level of constraint decreases with increasing distance from a gene.

## Methods

### Selection and amplification of human non-coding sequences

The sequences studied were in the region corresponding to chr4:41,443,127–41,579,542 in the human March 2006 (hg18) build. Using standard PCR conditions, sequences (Additional file 1 Table S1) were amplified off of human genomic DNA and separately subcloned into the pT2GWcfosEGFP vector, a Tol2-based transgenic reporter construct [19,29,39]. We, and others, have previously shown this to be a reliable screen for enhancer activity [15,19,39]. 333 base pairs were omitted from the tiling path due to primer design issues; the non-conserved amplicons were designed to have 0% overlap with the elements identified within the PhastCons Placental Mammal Conserved Elements, 28-way Multiz Alignment track [43].

### Fish care

All zebrafish were raised, bred, and staged according to standard protocols at 28°C [52,53] and under protocols approved by the Johns Hopkins University Animal Care and Use Committee.

### Embryo injections and analysis

Putative regulatory elements subcloned into the pT2GWcfosEGFP reporter construct were injected into wild-type G0 AB zebrafish embryos [39]. Reporter expression directed by each construct was then evaluated in ≥200 live G0 mosaic embryos at 24, 48, 72 and 96 hours post fertilization, requiring that consistent signal is observed among ≥10% of injected embryos. Analysis of embryos was conducted using a Carl Zeiss Lumar V12 Stereo-microscope with AxioVision version 4.5 software.

### Analysis of ENCODE data

#### Defining ENCODE regions for analysis

The human genome sequence (hg17) regions studied were obtained using the UCSC Genome Browser [54] based on ENCODE coordinates [25]. The corresponding 44 regions encompass 29,998,060 base pairs. From the Galaxy2ENCODE (GENCODE) data sets available on the Galaxy database, partitioned intervals representing the ENCODE region were obtained as a custom track ([55,56]; ). The ENCODE sequence data was partitioned into 7 sub-regions: coding sequence; 5' UTR; 3' UTR; intronic proximal (intronic ≤5 kb from an exon); intronic distal (intronic >5 kb from an exon); intergenic proximal, between genes ≤5 kb from an exon; and intergenic distal, between genes >5 kb from an exon [55]. The partitioned ENCODE region was composed of 10,689 regions representing 30,717,051 base pairs, 718,991 base pairs more than the region represented by UCSC Genome Browser defined ENCODE regions. Using the Galaxy Database Subtraction tool, we determined that 167,090 of the extra base pairs were accounted for by partitioned sub-regions that lay outside the UCSC genome browser defined regions, which were excluded from the analysis. Using the Galaxy Database Base Coverage tool, we determined that the rest of the remaining 551,901 excess base pairs arose from redundant partitioning of the same genomic intervals into multiple sub-regions. Manual curation of the region was used to remove the redundancy, leaving only 6,789 base pairs of overlap (0.02% of the ENCODE data set). The curated ENCODE partition sub-regions consisted of 10,052 intervals equaling 30,004,849 base pairs. The Galaxy Database Base Coverage tool was used to confirm that the manually curated ENCODE partitioned sub-regions had the same actual base coverage as the UCSC defined ENCODE regions (29,998,060 base pairs). To confirm the accuracy of our partitions the total base pairs in each sub-regions were summed, and were shown to be equal to the total base pairs in the manually curated ENCODE partitioned sub-regions.

#### Defining data sets for analysis

Using the UCSC Table Browser, the human genomic coordinates (hg18) for the PhastCons Placental Mammal Conserved Elements, 28-way Multiz Alignment, which are not available in hg17, overlapping the ENCODE region were downloaded as a custom track. The Galaxy Database Liftover Convert Genome Coordinates tool was used to convert the PhastCons Placental Mammal Conserved Elements, 28-way Multiz Alignment ([41-43]), genomic intervals from hg18 to hg17. These elements were then partitioned into ENCODE sub-regions using the Galaxy Database Gencode Partition tool. The total PhastCons Placental Mammal Conserved Elements, 28-way Multiz Alignment base pairs in each sub-region were then summed and termed conserved base pairs. The total non-conserved base pairs were then calculated by subtracting the conserved base pairs from each sub-region from the total base pairs for each sub-region (Additional file 1, Table S2).

To examine the effects of repeats on pTRR density, we calculated the amount of repeat DNA in each ENCODE sub-region. The hg17 repeat masker track for the UCSC defined ENCODE regions was downloaded in a custom track. To remove any overlap from repeat elements, the Galaxy Database Merge tool was used to concatenate overlapping repeat regions into single intervals. The merged repeats were then partitioned into ENCODE sub-regions using the Galaxy Database Gencode Partition tool. The total repeat base pairs were calculated for each sub-region using the merged partitioned repeats. To confirm that no repeats were lost in the Merge program, the Galaxy Database Base Coverage tool was used to confirm that the repeat masker and merged repeat intervals covered the same total genomic region (Additional file 1 Table S2).

To calculate the number of repeats that overlap with conserved regions, the Galaxy Database Intersection tool was used to calculate the intervals of base pair overlap between the PhastCons Placental Mammal Conserved Elements, 28-way Multiz Alignment intervals and merged repeat masker intervals. The regions of these overlaps were termed conserved repeats. The conserved repeats were then partitioned into ENCODE sub-regions using the Galaxy Database Gencode Partition tool. The total numbers of base pairs for each sub-region were summed. The conserved repeat base pairs were used to calculate the number of non-conserved repeat base pairs by subtracting the total number of conserved repeat base pairs from the number of total repeat base pairs for each sub-region. To calculate the non-conserved non-repeat base pairs, the non-conserved repeat base pairs were subtracted from the total non-conserved base pairs for each sub-region. To calculate the conserved non-repeat base pairs, the total conserved repeat base pairs were subtracted from the total conserved base pairs for each sub-region (Additional file 1 Table S2).

#### pTRR analysis

The hg 17 genomic coordinates for putative transcriptional regulatory regions identified by the ENCODE project [26] were obtained at . The UCSC Table Browser Intersection tool was then used to determine which pTRRs overlap the PhastCons Placental Mammal Conserved Elements, 28-way Multiz Alignment, termed conserved pTRRs, and those which do not, termed non-conserved pTRRs. The conserved and non-conserved pTRRs were then partitioned into the ENCODE sub-regions using the Galaxy Database Gencode Partition pool. The total number of conserved and non-conserved pTRRs for each sub-region was totaled (Table 3, Additional file 1 Table 2).

To calculate the density of pTRRs in each sub-region, the number of pTRRs in each sub-region was divided by the total number of base pairs in each sub-region, with a density of pTRRs per base pair. To calculate the density of conserved pTRRs in conserved regions, the total number of conserved pTRRs in each sub-region was divided by the total number of conserved base pairs in each sub-region. To calculate the density of non-conserved pTRRs, the number of non-conserved pTRRs in each sub-region was divided by the total number of non-conserved base pairs in each sub-region (Additional file 1 Table 2, Table 4).

To examine the effects of repeats on conserved pTRR density, the number of conserved pTRRs was divided by the number of conserved non-repeat base pairs in each sub-region. Also, the number of non-conserved pTRRs was divided by the number of non-conserved non-repeat base pairs in each sub-region (Additional file 1 Table 2).

#### Gene desert versus non-gene desert analysis

To study the effects of gene deserts on pTRR density, the gene desert regions of the ENCODE region were identified by manually curating the intergenic distal sub-regions to identify regions that had a minimum of 50 kb of overlap with a = 500 kb region that did not contain a National Center for Biotechnology Information Reference Sequence gene [50,51]. All intergenic distal gene desert regions were expanded 500 base pairs in the 3' and 5' direction; these expanded regions were then used on the UCSC Table Browser intersection tool to find all intergenic proximal regions flanking gene desert regions. The total number of base pairs, non-conserved base pairs, conserved base pairs, repeat base pairs, conserved repeat base pairs, non-conserved repeat base pairs, conserved non-repeat base pairs, non-conserved non-repeat base pairs, conserved pTRRs, and non-conserved pTRRs were calculated for both the gene desert intergenic distal and intergenic proximal regions as described above. The totals of the gene desert regions were then subtracted from intergenic proximal and intergenic distal all genes totals to calculate non-gene desert statistics. The density of conserved pTRRs, non-conserved pTRRs and the repeat adjusted density of conserved pTRRs and non-conserved pTRRs was then calculated as described above (Additional file 1 Table 4).

#### Analyses of Developmental versus Non-developmental Gene Intervals

To determine whether pTRR density differed between flanking developmental genes and non-developmental genes, Gene Ontology terms were used to classify the genes in the ENCODE region [48]. Using the UCSC Table Browser, the table for MGC genes in the ENCODE region was downloaded. Gene ontology term GO:0032502 was chosen as a reference to define developmental genes versus non-developmental genes. The gene names off the MGC gene table were then entered into the GO slimmer tool (; [49]), with the setting All species databases, All evidence codes, GO Slimmer term GO:0032502. Of the genes input into GO slimmer, 230 total unique genes were recognized by the database. Gene names not recognized by GO slimmer were excluded from the analysis. Of these, 78, labeled developmental genes, were labeled with the GO term GO:0032502, while 152 genes, termed non-developmental genes, were not labeled with GO:0032502. Manual curation was then used to create separate UCSC Genome Browser custom tracks containing the gene regions of the non-developmental and developmental genes respectively. These gene region intervals for both non-developmental and developmental genes were then expanded 7.5 kb in both the 5' and 3' direction, to allow identification of intergenic proximal and intergenic distal regions flanking the developmental and non-developmental regions. Using the UCSC Table Browser intersection tool, the overlap was found between the expanded developmental gene intervals and the intergenic proximal and intergenic distal sub-regions. Using the same method the overlap between the non-developmental gene expanded intervals and the intergenic proximal and intergenic distal sub-regions was also identified. To identify sub-regions that were positioned between a developmental gene and a non-developmental gene, and thus represented in both data sets, the UCSC Table Browser intersection tool was used to identify regions of overlap between the developmental gene intergenic proximal sub-regions and the non-developmental intergenic proximal sub-regions; and the overlap between the developmental gene intergenic proximal sub-regions and the non-developmental intergenic proximal sub-regions. These regions of overlap were excluded from the analysis due to their presence in both data sets. Using the UCSC Table Browser intersection tool the regions of overlap were subtracted from developmental and non-developmental gene intergenic proximal and intergenic distal sub-regions to leave four non-overlapping data sets termed developmental gene intergenic proximal non-overlap regions, developmental gene intergenic distal non-overlap regions, non-developmental gene intergenic proximal non-overlap regions, and non-developmental gene intergenic distal non-overlap regions. For these four data sets total number of base pairs, non-conserved base pairs, conserved base pairs, repeat base pairs, conserved repeat base pairs, non-conserved repeat base pairs, conserved non-repeat base pairs, non-conserved non-repeat base pairs, conserved pTRRs, and non-conserved pTRR and appropriate pTRR densities were calculated as above (Additional file 1 Table 5).

## Authors' contributions

ASM conceived this study. DMM and ZES performed analysis and imaging of zebrafish embryos. DMM, RMV and JH performed injections of zebrafish. DMM and ZES performed amplification and cloning of sequences. ZES performed analysis of ENCODE data. DMM, ZES, and ASM drafted manuscripts with revisions from JH and RMV. All authors read and approved by the final manuscript.

## Supplementary Material

Additional file 1 **Supplementary tables S1-S5** Click here for file
