# Supplementary material for: Asymmetrical distribution of non-conserved regulatory sequences at PHOX2B is reflected at the ENCODE loci and illuminates a possible genome-wide trend
Source: BMC Genomics. 2009 Jan 7;10:8. doi: 10.1186/1471-2164-10-8 (PMC2630312; doi:10.1186/1471-2164-10-8)
Supplement: Additional file 1 — Supplementary tables S1-S5 [file 1471-2164-10-8-S1.pdf]

Supplemental Tables

Amplicon	Coordinates (hg18)	Primer
PHOX2B-HNCS +1.7	chr4:41443126-41444037	F-AAGCAGGGGGAGAAAGAAGA<br>R-CTGGAGCCTCGAGTGAGAAG
PHOX2B-HCS +0.6	chr4:41444329-41445139	F-AGACAGAAAGTGAGCAAATCAGC<br>R-GGACCTTCAGCTTTCTCAGC
PHOX2B-HCS -0.0	chr4:41445738-41446286	F-GAATTTAATACCACACCATTAC<br>R-TTTTTAAGTGGCTGTATACCCT
PHOX2B-HCS -0.6	chr4:41446311-41446680	F-GCTTTCGAATGTGAAACATCTG<br>R-ACCCAAAAGCCTGAGGAATC
PHOX2B-HCS -0.9	chr4:41446705-41447657	F-CTCCCCTCCCCTTCTCTTC<br>R-AGGAGCGAGCAGTGATTTGT
PHOX2B-HCS -1.9	chr4:41447684-41448427	F-GCCCAGATCCCTTAGCTTCT<br>R-TTAAGGGAGGTGGGGGATAG
PHOX2B-HCS -2.9	chr4:41448641-41449895	F-CCTTCTCGCCTTTGTACCAG<br>R-TCTTTTTGGAGAAGCCCAAG
PHOX2B-HNCS -4.2	chr4:41449900-41450781	F-GTGGAGTGGGGCTTTGAAAT<br>R-CTGCAGACAGCAGCCTTTC
PHOX2B-HCS -4.9	chr4:41450785-41451074	F-CTCCCCTCCCCTTCTCTTC<br>R-AAGCTTGGTCAGGGCTCAT

PHOX2B-HNCS -5.3	chr4:41451079-41454153	F-TGAGACAAACAAACAGCAGCA<br>R-GGTGCAGAGGGGATTATGATT
PHOX2B-HCS -8.4	chr4:41454166-41456300	F-TGGGTGGATCATCTGAGGTC<br>R-TAGGAGGTTTGTGGGGTTTG
PHOX2B-HCS-34.8	chr4:41480567-41483210	F- GGGATTACAGGCATGATGTTTGGCTTT<br>R-TCTCTGCCATTTTTGCAGGCAATTT
PHOX2B-HNCS -71.5	chr4:41517243-41518675	F-TCCCTATTTGTAGGGGGAAGA<br>R-ACCAGGCAAGTGTCTGTTCA
PHOX2B-HCS -73.5	chr4:41519203-41519532	F-GCCTGGTGTCTGTGTCTCTG<br>R-TTCTCTTTCTCCCTCCCTGA
PHOX2B-HCS +106.1	chr4:41551816-41552776	F-AAGGGCCTACACCAATAGGG<br>R-CAAAAGGAGCACAACTAAACCTT
PHOX2B-HCS-107.2	chr4:41552948-41553287	F-TTCTGGGCACACTCTGTTTG<br>R-AGTTAGAGGCAGGAATATTAGAAACAT
PHOX2B-HCS -108.3	chr4:41554016-41554539	F-ATGGGGCAGGAAGGTTAAGT<br>R-TCCGCGTACCCAGAAAATAA
PHOX2B-HNCS -110.6	chr4:41556355-41556858	FGGGACTATATAAAGACAACATTTTTCA<br>R-CCATATACTTTGCACCGCTTG
PHOX2B-HNCS -112.3	chr4:41558009-41560428	F-TGCCCACAGGGTTCTCATTACAGC<br>R-GGACCCGGGTATTCCCTTCCA

PHOX2B-HCS -114.8	chr4:41560500-41560924	F-GAGGCAGCTGTGTGTGTTTG<br>R-CAGGTTTGTGCAGCCTTTTT
PHOX2B-HNCS -115.3	chr4:41561092-41562094	F-GTGTGTGCTGGTGAAATTGG<br>R-GGCTGAGGAGGTGTCTTCAC
PHOX2B-HCS -116.7	chr4:41562431-41562641	F-CACCGAATCCAAGTCTGTACTTT<br>R-CTGTGGTGAAGCCTGCATT
PHOX2B-HNCS -128.9	chr4:41573637-41574910	F-CGCCACCATACCCATCTAAT<br>R-CGCGGAAAATCTTTTGTTG
PHOX2B-HCS -130.4	chr4:41576125-41576259	F-CTCACCCCTGGGAAGACTC<br>R-TGGATATGAAGATTTGCCGATA
PHOX2B-HCS-133.5	chr4:41579270-41579542	F-TTCCAGACGGCTCTGACCT<br>R-CGGCCTATCCGAAATTACC

Supplemental Table S1. Primers and genomic coordinates (March 2006 build; hg18) of amplicons generated from human genomic DNA.
Amplicons named by their position relative to the transcriptional start site. F, forward primer; R, reverse primer.

Supplemental Table S2. Enumeration of pTRRs and base pairs of whole ENCODE sub-regions.

Distribution of putative transcriptional regulatory regions (pTRRs) partitioned into sub-regions from King et al. [26]. Conserved pTRRs are defined as regions overlapping the PhastCons Placental Mammal Conserved Elements, 28-way Multiz Alignment intervals [41, 43], non-conserved pTRRs defined as all other pTRRs. pTRRs were assigned to a partitioned region using Galaxy Gencode partition tool [55]. Base pairs in ENCODE partitioned sub-regions comes from summing up base pairs in each partitioned ENCODE defined region. Base pairs in conserved region is defined as sum of all genomic regions in the ENCODE region that are identified as PhastCons Placental Mammal Conserved Elements, 28-way Multiz Alignment intervals [43]. Non-conserved base pairs were calculated by subtracting conserved base pairs from total base pairs. Total repeat base pair was calculated by summing up the repeat masked base pairs in each sub-region. Conserved repeat base pairs were calculated from summing up the base pairs from the overlap of the repeat masker and PhastCons Placental Mammal Conserved Elements, 28-way Multiz Alignment intervals. The non-conserved repeat base pairs were calculated by subtracting conserved repeat base pairs from total repeat base pairs. The total conserved non-repeat base pairs were calculated by subtracting conserved repeat base pairs from conserved base pairs. The total non-conserved non-repeat base pairs were calculated by subtracting non-conserved repeat base pairs from non-conserved base pairs.

Supplemental Table S3. Enumeration of pTRRs and base pairs of Developmental and Non-developmental gene sub-regions.

Developmental genes were identified using GO Slimmer to filter for ENCODE region genes labeled with gene ontology term. GO:0032502. Non-developmental genes are those not labeled with GO:0032502. Distribution of putative transcriptional regulatory regions (pTRRs) partitioned into sub-regions from King et al. [26]. Conserved pTRRs are defined as regions overlapping the phastCons PhastCons Placental Mammal Conserved Elements, 28-way Multiz Alignment intervals [41, 43], non-conserved pTRRs defined as all other pTRRs. pTRRs were assigned to a partitioned region using Galaxy Gencode partition tool [55]. Base pairs in ENCODE partitioned sub-regions comes from summing up base pairs in each partitioned ENCODE defined region. Base pairs in conserved region is defined as sum of all genomic regions in the ENCODE region that are identified as PhastCons Placental Mammal Conserved Elements, 28-way Multiz Alignment intervals [43]. Non-conserved base pairs were calculated by subtracting conserved base pairs from total base pairs. Total repeat base pair was calculated by summing up the repeat masked base pairs in each sub-region. Conserved repeat base pairs were calculated from summing up the base pairs from the overlap of the repeat masker and PhastCons Placental Mammal Conserved Elements, 28-way Multiz Alignment intervals. The non-conserved repeat base pairs were

calculated by subtracting conserved repeat base pairs from total repeat base pairs. The total conserved non-repeat base pairs were calculated by subtracting conserved repeat base pairs from conserved base pairs. The total non-conserved non-repeat base pairs were calculated by subtracting non-conserved repeat base pairs from non-conserved base pairs.

Supplemental Table S4- Enumeration of pTRRs and base pairs of Gene Deserts and Non-gene desert ENCODE sub-regions.

Distribution of putative transcriptional regulatory regions (pTRRs) partitioned into sub-regions from King et al. [26]. Conserved pTRRs are defined as regions overlapping the PhastCons Placental Mammal Conserved Elements, 28-way Multiz Alignment intervals [41, 43], non-conserved pTRRs defined as all other pTRRs. pTRRs were assigned to a partitioned region using Galaxy Gencode partition tool [55]. Base pairs in ENCODE partitioned sub-regions comes from summing up base pairs in each partitioned ENCODE defined region. Base pairs in conserved region is defined as sum of all genomic regions in the ENCODE region that are identified as PhastCons Placental Mammal Conserved Elements, 28-way Multiz Alignment intervals [43]. Non-conserved base pairs were calculated by subtracting conserved base pairs from total base pairs. Total repeat base pair was calculated by summing up the repeat masked base pairs in each sub-region. Conserved repeat base pairs were calculated from summing up the base pairs from the overlap of the repeat masker and PhastCons Placental Mammal Conserved Elements, 28-way Multiz Alignment intervals. The non-conserved repeat base pairs were calculated by subtracting conserved repeat base pairs from total repeat base pairs. The total conserved non-repeat base pairs were calculated by subtracting conserved repeat base pairs from conserved base pairs. The total non-conserved non-repeat base pairs were calculated by subtracting non-conserved repeat base pairs from non-conserved base pairs.

Supplemental Table S2.

ENCODE Sub-regions Analyzed	Conserved pTRRs	Non-conserved pTRRs	Total bp	Conserved bp	Non-conserved bp	Total Repeat bp	Total Conserved Repeat bp	Total non-conserved repeat bp	Non-conserved non-repeat bp	Conserved Non-repeat bp
5' UTR	71	46	99,440	32,447	66,993	27,548	3,360	24,188	42,805	29,087
3' UTR	15	12	382,329	84,376	297,953	77,108	2,471	74,637	223,316	81,905
Intergenic Proximal	61	163	2,429,196	72,270	2,356,926	1,220,699	5,542	1,215,157	1,141,769	66,728
Intergenic Distal	48	171	11,055,834	361,325	10,694,509	5,648,729	21,845	5,626,884	5,067,625	339,480
Intronic Proximal	173	457	8,903,959	253,108	8,650,851	3,733,203	18,527	3,714,676	4,936,175	234,581
Intronic Distal	55	122	6,462,925	234,227	62,28,698	2,797,328	12,801	2,784,527	3,444,171	221,426
Coding sequences	0	0	671,166	546,622	124,544	36,082	5,076	31,006	93,538	541,546

Supplemental Table S3.

ENCODE Sub-regions Analyzed	Conserved pTRRs	Non-conserved pTRRs	Total bp	Conserved bp	Non-conserved bp	Total Repeat bp	Total Conserved Repeat bp	Total non-conserved repeat bp	Non-conserved non-repeat bp	Conserved Non-repeat bp
Developmental Intergenic Proximal	20	22	392,692	31,822	360,870	169,007	1,658	167,349	193,521	30,164
Developmental Intergenic Distal	5	20	1,636,075	56,850	1,579,225	626,673	3,282	623,391	955,834	53,568
Non-developmental Intergenic Proximal	24	86	733,487	20,809	712,678	402,526	1,102	401,424	311,254	19,707
Non-developmental Intergenic Distal	10	51	2,309,353	61,601	2,247,752	1,310,832	3,845	1,306,987	940,765	57,756

Supplemental Table S4

ENCODE Sub-regions Analyzed	Conserved pTRRs	Non-conserved pTRRs	Total bp	Conserved bp	Non-conserved bp	Total Repeat bp	Total Conserved Repeat bp	Total non-conserved repeat bp	Non-conserved non-repeat bp	Conserved Non-repeat bp
Non-Gene Desert Intergenic Distal Regions	39	159	7,147,316	206,965	6,940,351	3,925,643	13,771	3,911,872	3,028,479	193,194
Gene Deserts Intergenic Distal	9	12	3,908,518	154360	3,754,158	1,943,443	8,074	1,935,369	1,818,789	146,286
Gene Deserts Intergenic Proximal	0	3	75,000	6,529	68,471	33,785	276	33,509	41,491	6,253
Non-gene deserts intergenic proximal	61	160	2,354,196	65,741	2,288,455	1,186,914	5,266	1,181,648	1,100,278	60,475
